# Directional Selection from Host Plants Is a Major Force Driving Host Specificity in *Magnaporthe* Species

**DOI:** 10.1038/srep25591

**Published:** 2016-05-06

**Authors:** Zhenhui Zhong, Justice Norvienyeku, Meilian Chen, Jiandong Bao, Lianyu Lin, Liqiong Chen, Yahong Lin, Xiaoxian Wu, Zena Cai, Qi Zhang, Xiaoye Lin, Yonghe Hong, Jun Huang, Linghong Xu, Honghong Zhang, Long Chen, Wei Tang, Huakun Zheng, Xiaofeng Chen, Yanli Wang, Bi Lian, Liangsheng Zhang, Haibao Tang, Guodong Lu, Daniel J. Ebbole, Baohua Wang, Zonghua Wang

**Affiliations:** 1 Fujian-Taiwan Joint Center for Ecological Control of Crop Pests, Fujian Agriculture and Forestry University, Fuzhou, 350002, China; 2 Fujian University Key Laboratory for Functional Genomics of Plant Fungal Pathogens, Fujian Agriculture and Forestry University, Fuzhou, 350002, China; 3 Fujian Province Key Laboratory of Pathogenic Fungi and Mycotoxins, Fujian Agriculture and Forestry University, Fuzhou, 350002, China; 4 Haixia Institute of Science and Technology (HIST), Basic Forestry and Proteomics Research Center, Fujian Agriculture and Forestry University, Fuzhou, 350002, China; 5 State Key Laboratory Breeding Base for Zhejiang Sustainable Pest and Disease Control, Institute of Plant Protection Microbiology, Zhejiang Academy of Agricultural Sciences, Hangzhou, 310021, China; 6 Haixia Institute of Science and Technology (HIST), Center for Genomics and Biotechnology, Fujian Agriculture and Forestry University, Fuzhou, 350002, China; 7 Department of Plant Pathology and Microbiology, Texas A&M University, College Station, TX, USA

## Abstract

One major threat to global food security that requires immediate attention, is the increasing incidence of host shift and host expansion in growing number of pathogenic fungi and emergence of new pathogens. The threat is more alarming because, yield quality and quantity improvement efforts are encouraging the cultivation of uniform plants with low genetic diversity that are increasingly susceptible to emerging pathogens. However, the influence of host genome differentiation on pathogen genome differentiation and its contribution to emergence and adaptability is still obscure. Here, we compared genome sequence of 6 isolates of *Magnaporthe* species obtained from three different host plants. We demonstrated the evolutionary relationship between *Magnaporthe* species and the influence of host differentiation on pathogens. Phylogenetic analysis showed that evolution of pathogen directly corresponds with host divergence, suggesting that host-pathogen interaction has led to co-evolution. Furthermore, we identified an asymmetric selection pressure on *Magnaporthe* species. *Oryza sativa*-infecting isolates showed higher directional selection from host and subsequently tends to lower the genetic diversity in its genome. We concluded that, frequent gene loss or gain, new transposon acquisition and sequence divergence are host adaptability mechanisms for *Magnaporthe* species, and this coevolution processes is greatly driven by directional selection from host plants.

Present knowledge regarding pathogen-host interaction shows that, some plant pathogens have a broad host range and are capable of parasitizing host plants of different families. In contrast, the other group of plant pathogens are described as host specific because their parasitic activities are limited to specific plant species or specific plant families^1^,^2^. In spite of the host specificity of host specific pathogens, host jump and host expansion are common evolutionary mechanisms in plant pathogens and enables host specific pathogens to shift from one host to another or acquire new host and in most cases, host shift consequently produces the most devastating disease outbreaks^3^. Previous findings revealed that speciation following host jump determines the success with which pathogens can adapt and survive in their new host^4^. In the speciation process, the genome undergoes great changes that allow the pathogen to acclimatize with the environment of their new hosts^5^,^6^,^7^,^8^. However, the speciation process is largely influenced by the incompatibility between pathogen and potential host plants. Domestication of plants promotes significant morphological and genetic changes which in turn greatly reduces genetic diversity of the host plants and that of pathogens^9^,^10^.

Rice constitutes the main source of calories for about 30% of the world’s population and represents a major food crop that contributes significantly towards the realization of household, national, regional and global food security^11^. Rice was domesticated from the grass family, which comprises of more than 10,000 plant species^12^,^13^. However, one principal factor undermining rice cultivation worldwide is the rice blast disease inflicted by an Ascomycota fungus *Magnaporthe oryzae*. In view of the fact that, *M. oryzae* infection often results in significant yield losses and accounts for an annual yield loss of about 18% worldwide makes the blast disease an important threat to food security worldwide^14^,^15^. Investigations showed that besides rice, *Magnaporthe* species is also capable of causing blast disease on more than 50 plant species of monocot origin including food crops namely; wheat, millet, and barley. In addition, *Magnaporthe* species also infects wild grass hosts such as *Digitaria sanguinalis*, *Setaria viridis*, and *Eleusine indica*^16^. Currently, *M. oryzae* and *M. grisea* are the most studied members of the *Magnaporthe* species because they are highly amenable and have undergone rapid co-evolutionary change that allows them to parasitize new hosts. The emergence of wheat blast disease in Brazil is relatively new and does not only demonstrate host jump and host expansion capabilities of *Magnaporthe* species, but also poses great challenge to researchers^17^.

Furthermore, it has been acknowledged that, secreted proteins potently induce or suppress plant immunity and also facilitate frequent jump of transposons and subsequently promote host jump in *M. oryzae*^18^,^19^. For instance, the secreted effector PWL2, a host specificity determinant, acting much like an avirulent gene, was cloned from rice isolates and the presence of this gene potently boosted the resistance of weeping love grass (*Eragrostis curvula*) against being infected by rice isolates^20^. *AVR1-CO39* cloned from grass isolate 2539 confers avirulence toward rice and there is no complete *AVR1-CO39* in rice isolates^21^,^22^,^23^. Although, *M. grisea* and *M. oryzae* are closely related from genetic and phylogenetic point of view, many of their effector proteins are however unique to specific species and show no evolutionary conservation beyond a narrow group of closely related populations. Moreover, there are indications that effector proteins might be controlling host specificity in *Magnaporthe* species. Available literature indicates that, in *M. oryzae*, transposons can affect the expression of some avirulent genes that mediates effector triggered immunity in plants and hence enable the rice blast fungi to avoid the recognition mechanisms of host plant’s immunity system^18^,^19^,^24^,^25^,^26^,^27^,^28^.

Numerous genetic analysis conducted with multi-locus approach has illustrated how two members of the *Magnaporthe* species; *M. grisea* and *M. oryzae* differ from each other on the basis of host preference. Such studies has effectively proven that*, M. grisea* only infect *Digitaria genera*, whilst, *M. oryzae* comprises of all isolates associated with rice and other grass species^29^,^30^. The taxonomic relationship between *M. grisea*, *M. oryzae* and its Sordariomycetidae fungi have been well studied using data generated from morphological, phylogenetic and genomic studies^31^,^32^,^33^. However, how genome evolution of *M. grisea* and *M. oryzae*, especially the evolution process for the host specificity has not been carefully studied yet. In this study, we carried out comparative genomic study on the two closely related *Magnaporthe* species, *M. grisea* and *M. oryzae* in line with the conviction that genome-wide study of isolates from different hosts will provide additional and useful information that will facilitate pursuits of understanding the genomic characteristics that underlying host speciation and the genomic differentiation of pathogen associated with the process of domestication of host plants.

## Results

### Host specificity of *Magnaporthe* species

**Figure 1:**
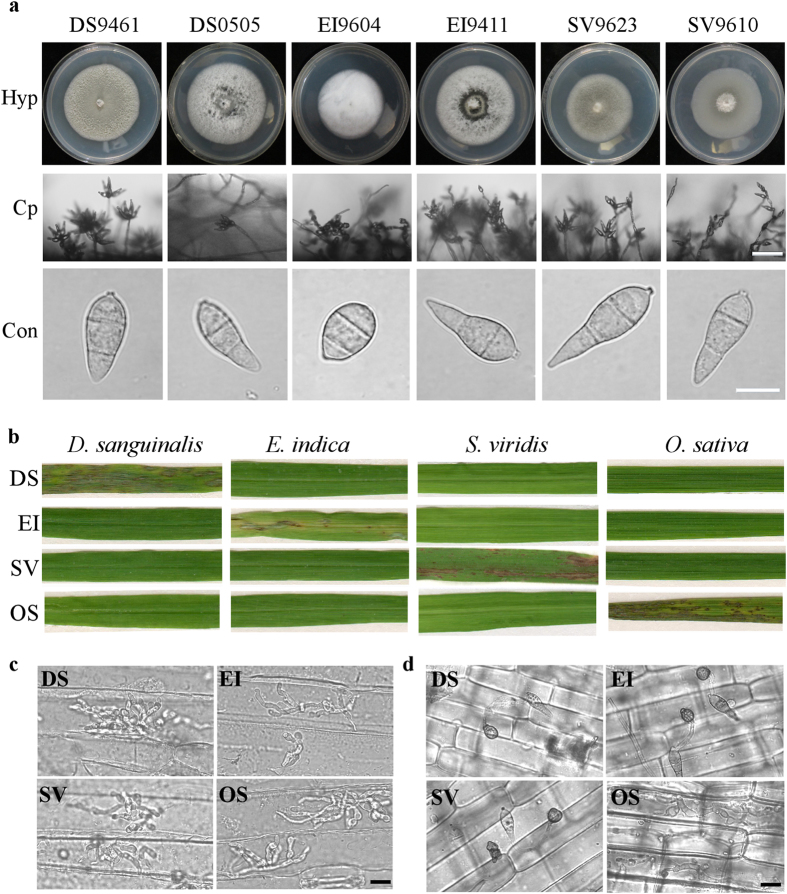
Host specificity of *Magnaporthe* species. (**a**) Hyphae (Hyp), conidiophore (Cp) and conidium (Con) morphology of *Magnaporthe* species isolates. Bar = 50 μm (conidiophore) and 10 μm (conidia). (**b**) Pathogenicity assay of *Digitaria sanguinalis* isolates (DS), *Eleusine indica* isolates (EI), *Setaria viridis* isolates (SV) and *Oryza sativa* isolates (OS) on *D. sanguinalis*, *E. indica*, *S. viridis* and *O. sativa*. (**c**) Barely leaf inoculation assay of tested isolates with conidial suspensions (1 × 10^5^ conidia/mL), and infectious growth was observed at 36 hpi. Bar = 10 μm. (**d**) Rice sheath inoculation assay of tested isolates with conidial suspensions (1 × 10^5^ conidia/mL), and infectious growth was observed at 48 hpi. Bar = 10 μm.

To ascertain the possible existence of host jump, host expansion or host tracking in known or identified host specific *Magnaporthe* species isolates that were deployed in this comparative genomic studies, we have investigated morphology and pathogenicity of *Magnaporthe* isolates from different host plants. We categorized and designated isolates to reflect the host they are associated with as; *D. sanguinalis* (DS), *E. indica* (EI), *S. viridis (*SV) and *O. sativa (*OS). From morphological characterization of *Magnaporthe* species isolates indicate these isolates produce morphologically indistinguishable hyphae, conidiophore and conidia (Fig. 1a). Multiple crossed pathogenicity assay was carried-out with conidia harvested from respective isolates for all of the isolates and one of representative result have been presented. As shown in (Fig. 1b), the isolates investigated in this study retained their pathogenicity and were only pathogenic to their respective hosts and reaffirmed that the isolates are host specific.

To further understand what potential factors limit the infection abilities of isolates, we inoculated barley leaves and rice sheaths with conidial suspensions. The results showed that barley is highly susceptible to all *Magnaporthe* isolates inoculated (Fig. 1c), but rice is only infected by isolates from *O. sativa* (Fig. 1d), indicating the host specificity is regulated by the differential recognition in immunity of different host-*Magnaporthe* pathosystem, not by the morphological or physical difference including the hardness and hydrophobicity of the surface between different host plants.

### Genome sequencing and assembly

Two isolates each from *D. sanguinalis*, *E. indica* and *S. viridis* were sequenced, and isolate names, host species are shown in Table 1. Some of the published genome sequences of isolates from *O. sativa* were also used to analyze in this study^34^,^35^,^36^,^37^. On average, 2.5 GB of raw reads each for all isolates were generated, which represent ~60 folds of sequencing depth, assuming a genome size of 40 Mb. *De-novo* assembly was performed and the genome assembly results were shown in Table 1. The draft genome of all isolates indicates the sizes of different isolate groups varied from each other by ~5 Mb (~12.5%). The GC content of the two *D. sanguinalis* isolates was 48.7% and 48.6%, respectively, which is lower than the values obtained for other isolates from *O. sativa* reported in previous investigations^34^,^35^,^36^,^37^.

The gene prediction was conducted using a combination of evidence-based and *ab initio* prediction. The number of predicted genes, average gene length and total percentage of coding region of assembly for each sequenced isolates are shown in Table 1. The contig number of isolates from *D. sanguinalis* and *E. indica* along with observed variations. In summary, the number of predicted genes, average gene length and percentage of coding region of the genome for isolates from the same host are very similar to each other, indicated a close relationship between isolates of host-specific populations.

### Phylogenetic analysis of *Magnaporthe* species

**Figure 2:**
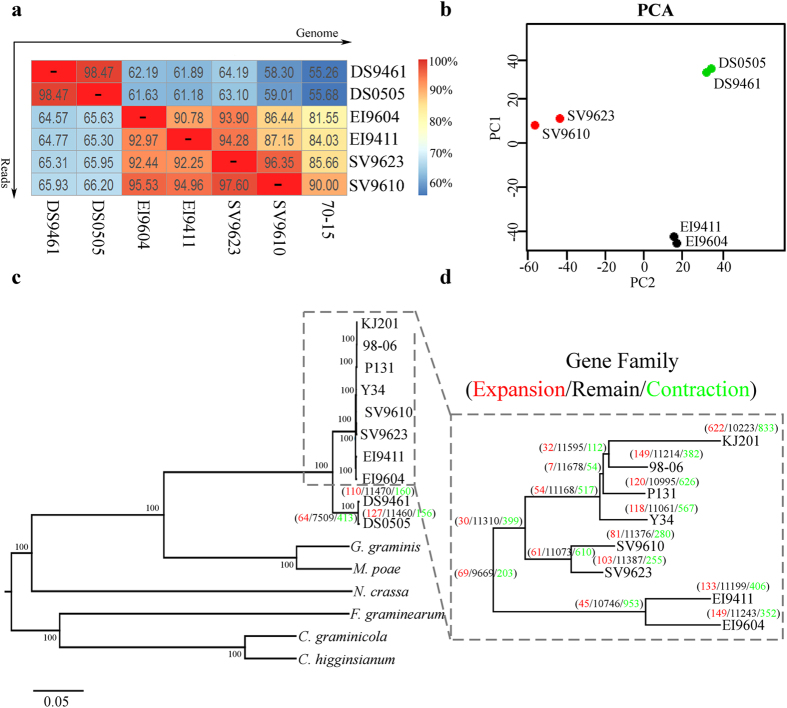
Genome reads mapping, principal components analysis (PCA), phylogenetic relationship and gene family comparison of *Magnaporthe* species. (**a**) The sequenced reads (vertical line) were mapped to genome (horizontal line), and the numbers in each blank represent the percentage of reads marked in vertical line that can be mapped into the genome marked in horizontal line. (**b**) Principal components analysis (PCA) of *Magnaporthe* species. (**c**) The phylogenetic tree based on amino acid sequences of 1693 single orthologous genes that exist in *Colletotrichum graminicola*, *Colletotrichum higginsianum*, *Fusarium graminearum*, *Gaeumannomyces graminis*, *Magnaporthe poae*, *Neurospora crassa* and *Magnaporthe* species isolates. (**d**) Enlargement of phylogenetic tree of *Magnaporthe* species isolates. The number of gene family under expansion (red), remain (black) and contraction (green) are indicated along the branch or node in the tree.

To evaluate the genomic relationship of different isolates, pair-wise genome comparison was conducted. The percentage of reads that mapped to the genome of isolate 70-15 and our sequenced isolates have been calculated in efforts to establish the direct differences between genomes (Fig. 2a). The genome difference was the highest between isolates from *D. sanguinalis* and other host species, ranging from 33.8 to 44.32%. Isolates from the same host were more similar to each other than isolates infecting other host genera. Principal Component Analysis (PCA) based on 46,530 SNPs obtained from 6 sequenced isolates also indicates three groups as the most likely partition of the 6 samples, corresponding to each of the three host plants (Fig. 2b).

To reconstruct the evolutionary history of *Magnaporthe* species and its relative Sordariomycetidae fungi, we selected *Colletotrichum graminicola*, *Colletotrichum higginsianum*, *Fusarium graminearum*, *Gaeumannomyces graminis*, *Magnaporthe poae* and *Neurospora crassa* to construct the phylogenomic tree. A total of 1693 single copy genes are present and conserved in selected genomes. As shown in (Fig. 2c), although there is an obvious divergence between *D. sanguinalis* isolates and other isolates, they still could be categorized into the same paraphyletic clade in comparison with *G. graminis*, *M. poae* and other fungi. To have a better view of the phylogenetic relationship of sequenced isolates, the tree branch containing *E. indica*, *S. viridis* and *O. sativa* isolates have been illustrated specifically, as shown in (Fig. 2d), there is a clear separation between isolates from different host plants where each host group forms a monophyletic clade. These results revealed that the phylogenetic relationship of *Magnaporthe* species isolated from the same host shares common phylogenetic history and implies that, they may as well utilize common host adaptation mechanisms.

### Comparative genomic analysis of *Magnaporthe* species

**Figure 3:**
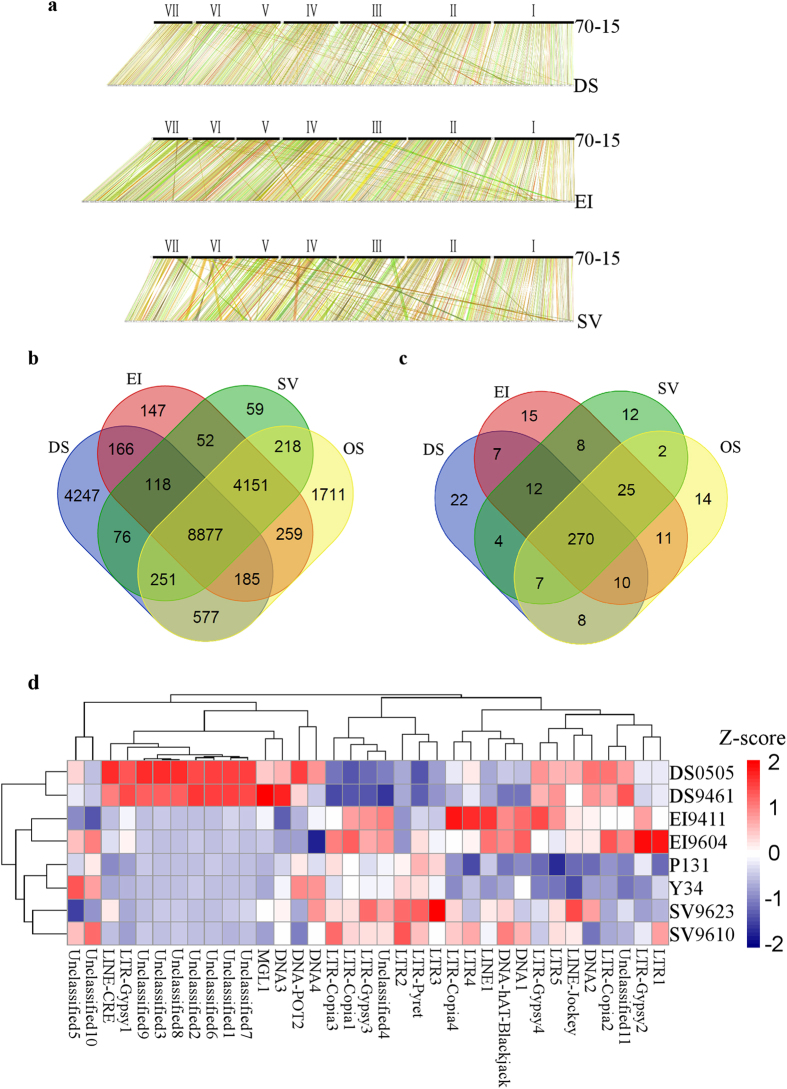
Comparative genomic analysis of *Magnaporthe* species. (**a**) Whole genome synteny comparison of isolates from different host plants with *M. oryzae* isolates, 70-15. (**b**) Venn diagrams shows unique genes belonging to isolates from different host plants. (**c**) Venn diagrams displays transposable elements identified in isolates from different host plants. (**d**) Hierarchical clustering analysis of copy number variation of the most abundant 35 kinds of transposable elements belonging to isolates from different host plants. Z-scores present variation of copy number with red color means increased number of transposon element and navy blue color means decreased number of transposon element. The copy numbers of transposon elements in each isolates are shown in Supplementary Table S3. DS, *D. sanguinalis* isolates, EI, *E. indica* isolates, SV, *S. viridis* isolates and OS, *O. sativa* isolates.

To gain insight into the genetic differentiation of isolates resulting from host specialization, we performed whole genome collinear analysis of sequenced isolates with 70-15. The synteny between orthologous gene of isolates and 70-15 show high density of genome collinear (Fig. 3a). Meanwhile, pair-wise comparisons of genes have also been conducted to identify overlapping and differentiations between isolates from different host. As shown in (Fig. 3b), all the four categories of isolates have 8,877 genes in common, comprising ~60% of genes of each isolates. However, it emerged from our analysis that, 3245, 274, 154 and 294 genes are unique to DS, EI, SV and OS groups respectively. The unique genes and their predicted function are shown in Supplementary Table S1. The functional prediction of these group unique genes revealed that cytochrome P450 gene family members constitutes the most abundant genes identified as unique genes.

To further understand the genome evolution as a function of host specialization, we assessed possible expansion of gene families based on the similarity of amino acid sequences (Fig. 2c,d). From the above investigation, we noticed that, the incidence of gene expansion rarely occurred among the various gene families. At the same time, we observed the occurrence of gene duplication among the isolates and identified 82 groups of genes with duplication in at least two isolates. However, the biological functions of most of these duplicated genes are unknown (Supplementary Table S2). Among these genes, we identified some genes with a duplication event after host speciation, such as MGG_16553 and MGG_15793, which have two copies in all *O. sativa* isolates, while no corresponding homologues in isolates from other host plants. Earlier studies indicated that these two genes are telomere-linked RecQ helicase (TLH) genes that play an important role in DNA repair and telomere maintenance^38^,^39^,^40^,^41^.

To characterize the evolution and dynamics of TE in *Magnaporthe* species, two *de novo* TE annotation methods have been used in this study. The two-tiered method provides a higher sensitivity in the identification of TE elements at both reads and genome levels. We found that our sequences contained all the published TE sequences and telemetric region repeat sequences in *M. oryzae*^42^. This allowed us to define the type and copy number of different TEs that varied significantly among different category of isolates. The TE types in the different isolates were identified with the customized TE library using the RepeatMasker as shown in (Fig. 3c). The total percentage of TE varies between the different isolates with the LTR family of TE showing the greatest variation, suggesting that the LTR elements may be differentially proliferated (Supplementary Table S3). To show the differentiation of TE in different isolates, we selected 35 TEs that are most abundant in isolates with similar genome assembled quality (Supplementary Table S4). As shown in (Fig. 3d), the copy numbers of these genome rich TEs varies significantly between different isolates from different host plant. For example, a LTR-Gypsy1 and some unclassified repeat elements were highly abundant in DS groups, whilst the quantity observed in other groups were significantly less. The high incidence of frequent gene loss, gain, acquisition of new transposon and sequence divergence as observed from our comparative genomic studies on the evolutionary processes that varies isolates of *Magnaporthe* species are subjected to further confirmed that, host specificity is rigidly regulated phenomenon dictated by their respective host plants.

### Directional natural selections in different groups of isolates

**Figure 4:**
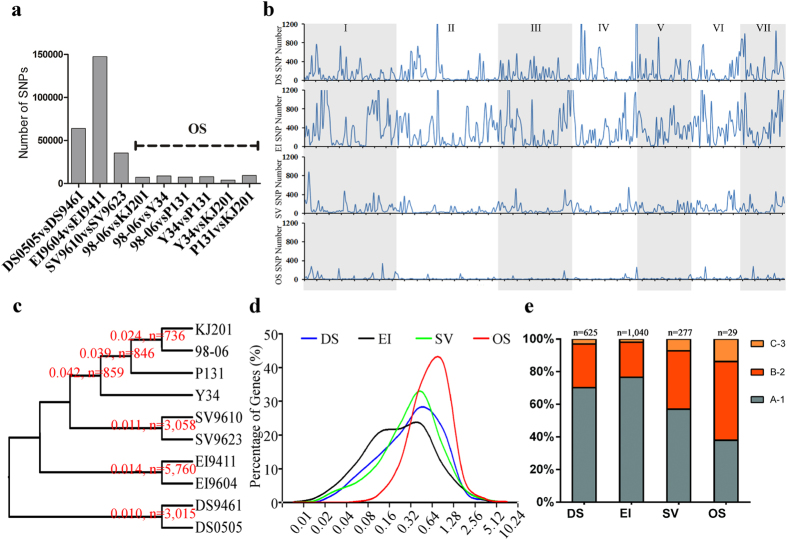
Whole genome comparison of natural selection between isolates belonging to the same host plants. (**a**) Inter-groups genomic comparison of SNPs number. X-axis represents isolates that have been compared and Y-axis represents total number of SNPs between compared genome. (**b**) Whole genome distribution of SNPs in different chromosomes. X-axis represents different chromosomes of reference genome 70-15 and Y-axis represents number of SNPs per 100 Kb. (**c**) Inter-groups comparison of nucleotide diversity (π). The number of nucleotide diversity (π), number of gene sets with π > 0 are indicated along the node in the tree. (**d**) The percentage of genes experienced different level of natural selection (KaKs). X-axis represents values of KaKs and Y-axis represents percentage of genes with corresponding KaKs value. (**e**) Showed overlapping genes identified under selection in four groups, A-1 represents genes only understand selection in one group, B-2 represents genes under selection in two groups and C-3 represents genes under selection in three groups. DS, *D. sanguinalis* isolates, EI, *E. indica* isolates, SV, *S. viridis* isolates and OS, *O. sativa* isolates.

To gain an insight into the natural selection of *Magnaporthe* associated with different host plants, whole genome SNPs comparison have been conducted to reflect the genomic diversity in different populations. The results of intra-group’s genome to genome comparison are shown in (Fig. 4a,b), from which we could see that the number of SNP are 63879, 147423, 35491 and 7449 (OS average), and the average SNP density in chromosomes are 147, 358, 91 and 23 per 100 Kb, for DS, EI, SV and OS, respectively. The dramatically decreased numbers of SNPs indicate the great loss of genetic diversity (“genetic sweep”) in *O. sativa* isolates. At the same time, we analyzed nucleotide diversity (π) and KaKs value of gene’s coding sequences. The gene sets of different isolates have been paired by bidirectional BLAST, and only genes that are reciprocal best hits (RBH) have been regarded as gene pairs. The number of RBH pairs in DS, EI, SV and OS groups are 11324, 12432, 12770 and 11085, respectively. The results established that although the number of genetic diversity sites (n) decreased in OS population (n = 813 for OS vs. n = 3015 for DS, n = 5760 for EI and n = 3058 for SV), interestingly, the average nucleotide diversity (π) is almost tripled (π = 0.035 for OS vs. π = 0.010 for DS, π = 0.014 for EI and π = 0.011 for SV) (Fig. 4c). The genes under selection (KaKs≠0) also changed greatly intra different groups. The number of genes under selection in DS, EI and SV are 1502, 2726 and 835, respectively, while this number in OS groups are 181. The average KaKs value of gene under selection are 0.481, 0.374, 0.464 and 0.719 (n = 1502, 2726, 835 and 181) for DS, EI, SV and OS, respectively. The peak of KaKs value increased from 0.08 ~ 0.64 to 0.64 ~ 1.28 for OS group (Fig. 4d). It is also noticeable that the KaKs values of inter-groups comparisons also show more selection sites and lower values (Supplementary Fig. S1). We also compared whether these are an overlap of genes that exist in all isolates as presented in (Fig. 3a). As shown in (Fig. 4e), the percentage of genes under selection in only one groups are much higher than genes under selection in two or three groups, and we did not find genes understand selection in four groups. These results proved that the genetic diversity of *Magnaporthe* isolates of *O. sativa* is under stronger host directed selection, and different groups of isolates under different selection closely related with their host.

### Evolution of secreted proteins under host directed selection

**Figure 5:**
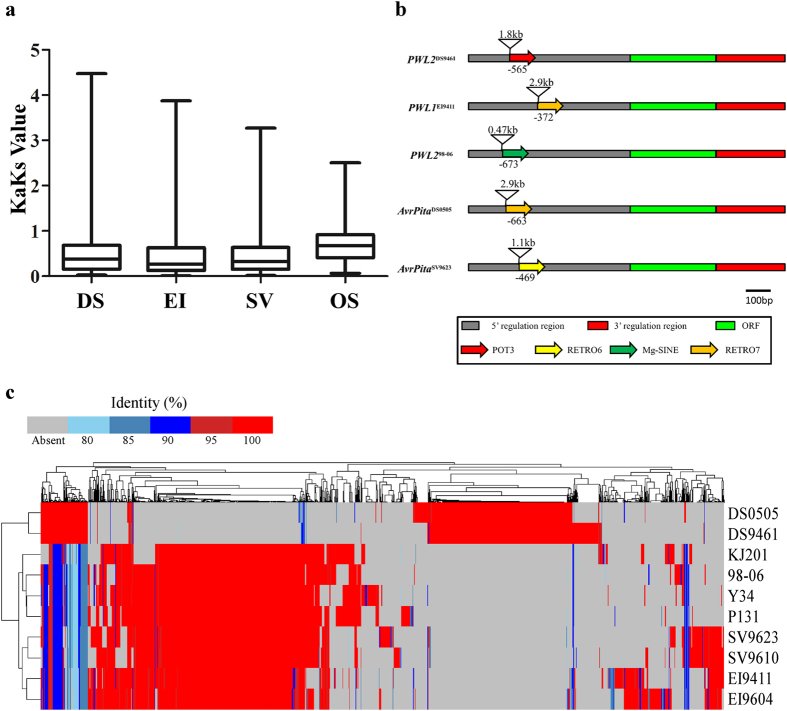
Whole genome comparison of secreted proteins. (**a**) The KaKs values of small secreted proteins. (**b**) Portrays incidence of transposon elements insertion in promoter region of *PWL1*, *PWL2* and *AvrPita* genes. (**c**) Hierarchical clustering analysis of Presence and Absence Variation (PAV) and amino acid identity of secreted proteins. As presented in the bar, grey means the absence of a gene and different colors represent corresponding identity of compared proteins. DS, *D. sanguinalis* isolates, EI, *E. indica* isolates, SV, *S. viridis* isolates and OS, *O. sativa* isolates.

Since secreted proteins play vital roles in plant-pathogen interaction, we decided to take inventory of secreted proteins in the whole genome sequence of all the isolates deployed in our investigation. In accordance with this objective, we identified a total of 11951 putative secreted proteins in the whole genome sequence of 10 isolates (Supplementary Table S5) and from our results we observed that the KaKs values of small secreted proteins in OS isolates are higher than other groups (Fig. 5a), which is 0.543, 0.547, 0.521 and 0.706 for DS, EI, SV and OS group, respectively. We further searched for the Presence and Absence Variation (PAV) within these secreted proteins (Fig. 5c). This examination showed that, lots of new secreted proteins have evolved in different isolates and interestingly some of these secreted proteins are only present in group of isolates from the same host plant. In addition, we observed that secreted proteins present in the same group exhibited high identity, suggesting a recent proliferation perhaps as adaptive response in the new host environment. For instance, we deployed PAV of avirulent (AVR) genes to show the existence of correlation between the evolution of AVR genes and host divergence (Table 2). Nevertheless, as shown in Table 2, nucleotide diversity (π) observed among AVR genes from the different isolates are lower than expected. Apart from the observed PAV, we also identified point and insertional mutations that are unique to specific host plants. While, we also found a strong selection of AVR genes between isolates from different host plants. For example, *AvrPiz-t* (KaKs = 1.53 between OS and EI, under strong positive selection) in two *Elusine* isolates has 3 non-synonymous point mutation at the same sites, and *AvrPi9* (KaKs = 0, under strong purifying selection) in two *Elusine* isolates has the same 18 bp insertion and a nucleotide substitution in its intron region (supplementary Fig. S2 a,b). Furthermore, we also monitored the occurrence of transposon element insertion in the promoter region of all known AVR genes in 10 isolates of *Magnaporthe* species. From this investigation we identified 5 cases of transposon insertion in *PWL1*, *PWL2* and *AvrPita* with *PWL2* and *AvrPita* recording 2 insertion at different loci (Fig. 5b). These observations indicate that analysis of PAV and KaKs values are important tools to identify genes that may be directly involved in the host adaptation. However, it is important to note that known *AVR* genes may face different selection pressure in different hosts. In the case of *AvrPi9*, the lack of coding sequence diversity suggests the gene may be important for pathogenesis but not specific adaptation to a particular host.

## Discussion

Fungi contribute significantly to the sustainability of diverse ecosystems. They are heterotrophs and survive as saprophytic or symbiotic organisms and can be parasites of plants, animals or of other fungi. To achieve this, fungi have evolved sophisticated morphological structures coupled with flexible genomes. Fungal genome sizes could vary from ten Mb to few hundred Mb, the percentage of repeat sequences could also vary in magnitudes up to 60% or more. Accumulating evidence showed that new genes are continuously evolving in parasitic fungi and many of these genes are either pathogenicity genes or genes related to secondary metabolism^43^,^44^,^45^,^46^. These characteristic features, promotes the fungus kingdom as the most diversified kingdom in nature. More so, their heterotrophilic life style makes their evolution to be greatly influenced by their corresponding environment and hosts, which often lead to fascinating coevolution between their genomes. In this study, we conducted morphological and genomic comparison of different isolates of *Magnaporthe* species with different host preferences to illustrate the evolutionary processes of pathogens under the influence of selection exerted by different host plants.

From our pathogenicity trials with all the four categories of isolates examined, it is obvious that all the isolates are pathogenic to their respective known host, but were entirely non-pathogenic on non-host plants. This observation, coupled with the fact that these isolates produces morphologically indistinguishable hyphae, coloration and conidia. Evidently showed that, host specificity and adaptation of different pathotypes of *Magnaporthe* are not influenced by inherent morphological structures, but rather opined that host specificity and adaptation are evolutionary traits acquired by *Magnaporthe* species under strong selection pressures predetermined by host plants.

Since our earliest investigation established that, isolates derived from different host plants possessed morphologically indistinguishable features. We deemed it prudent to examine possible variations within the genome that might correspond with the host specificity traits of these isolates using comparative genomic study. In contrast to morphological data, we identified variation within the genome of these *Magnaporthe* species. The differences existing between genomes of these species are as high as 44%, in spite of these huge inter-genomic variations observed within these isolates. They nonetheless, remained members of the two well classified and studied groups *M. grisea* and *M. oryzae*, according to comparison studies conducted with closely related filamentous fungi Sordariomycetes^33^. *M. grisea*, which consists of isolates that are solely pathogenic to *D. sanguinalis* showed distant phylogenetic relationship with other group members of *Magnaporthe* species and hence, constitute an independent group of the *Magnaporthe* genus^29^,^30^.

Furthermore, our results showed that, incidence of genome differentiation has resulted in the generation of solitary group of genes which we referred to as lineage-specific genes. Additional functional predictions carried-out on these lineage-specific genes showed that, these genes play multiple biological functions and subsequently demonstrates that, rapid genome evolution associated with isolates represents an acquired biological transformation developed in response to host speciation. Among these lineage-specific genes, cytochrome P450 gene family members constitutes the most abundant genes influenced by genomic differentiation. Since cytochrome P450 family of genes play crucial role in the biosynthesis of secondary metabolites that invariably contribute to the virulence of pathogens and as well functions in the detoxification of phytoalexins generated by host plants in response to pathogen invasion. We therefore proposed the evolution of P450 gene family members may reflect the differentiations of pathogenicity and virulence while pathogens experience the host adaptation process^47^. More so, the high variations associated with the genome features of these isolates which belongs to the *Magnaporthe* species complex is an ample indication that, the blast pathogen experiences series of changes at genome level in order to condition it to fit enough for host speciation.

Our analysis depicts significant variation in the types and extent of Transposon Elements (TE) duplication between the different groups of isolates, thereby indicating that TEs play an important role in genome evolution and could be regarded as an important element responsible for genome differentiation in *Magnaporthe* species. Other studies conducted in *M. oryzae* revealed that transposons can influence the expression of some avirulent genes that mediates the effectors triggered immunity of plants and subsequently enabling the pathogen to avoid the recognition mechanisms of the host plant immune system^18^,^19^,^24^,^25^,^26^,^27^,^28^ and having realized that variations identified in the quantum of common TEs present in the isolates corresponds to their host preference and specificity. We concluded that, acquisition of TEs and overall manipulation of TEs in terms of copy numbers and positions might constitute host jump and host tracking mechanisms in *Magnaporthe*^30^. However, the factors that directly influences the copy numbers of TEs in *Magnaporthe* species prior to, during or following speciation still remains obscure and needs to be investigated in further research endeavors.

Supplementary results obtained by conducting comparison analysis with sequence obtained from the various isolates in reference to their host plants showed that *Magnaporthe* genome has been greatly influenced by host directed selection pressure. It also emerged that, host direct selection constitutes the main driving force that accelerates further differentiation of the *Magnaporthe* population. This finding although somehow ambivalent, still provided us with substantial clues suggesting that, *Magnaporthe* species as well as other plant pathogens can swiftly change their host preference. We evaluated the phenomenon with respect to time and under the limelight of host-pathogen coevolution, we positioned that, genome variability contributes to host jump in the short-term and are of the view that host jump could be substantially influenced by non-host resistance of plants. It is worthwhile mentioning here that, population of *Magnaporthe* species that are adapted to different host plants has experienced natural selection in varying intensities and in different directions. With the background that knowledge genetic diversity of plants greatly decreased with the domestication to meet the human’s needs^48^. It was therefore adequate to infer that the different levels of natural selection associated with the isolates are driven by the genetic diversity of host plants. We also asserted that different host-pathogen interaction mechanisms would be at play in other to foster successful parasitic relationship between these isolates and their respective host plants and subsequently concluded that, the differences in the direction of selection as observed between the isolates are driven by variations in host-pathogen interaction mechanisms^49^. More so, the process of domestication of plants under artificial selection practices could produce selective sweep on genome and result in low genetic diversity at some loci and should have exerted minimum selection pressure on the pathogens. However, because resistant genes are continuously introduced to domesticated plants, they tend to highly enriched in resistant genes that are readily deployed in response to biotic stresses^50^,^51^,^52^,^53^,^54^. These genetic manipulations constitute a major host genetic parameter exerting higher selection pressure on the pathogens and promoting directional selection^55^. In our comparative genomic studies carried-out on *Magnaporthe* isolates from *O. sativa* which is highly domesticated crop and *Magnaporthe* isolates from *D. sanguinalis*, *E. indica* and *S. viridis* which are undomesticated grasses, we have showed that, *Magnaporthe* isolates from domesticated *O. sativa* experienced a higher level of natural selection and displayed lower level of genetic diversity compared with *Magnaporthe* isolates sampled from wild plants; *D. sanguinalis*, *E. indica* and *S. viridis*. The distinct natural selection behavior of host plants on pathogen suggest that the domestication process of plants under artificial selection can produce selective sweeps on both the host plant and pathogen genomes. The above results has given us enough iota to conclude that, gain or loss of genes, acquisition of new transposable elements and sequence divergence driven by directional selection from host plants constitutes the principal factors driving host jump, host tracking, host expansion and host speciation in *Magnaporthe* species.

## Methods

### Isolates collection, isolates cultivation, pathogenicity assays, DNA isolation and genome sequencing

Isolates in this study was collected in field as: DS9461 and EI9411 were collected in Fujian province, the People’s Republic of China in 1994. EI9604, SV9610 and SV9623 were collected in Zhejiang province, PRC in 1996. DS0505 was collected in Zhejiang province, PRC in 2005. All the isolates were cultured at 26 °C 10 days to take photo using complete medium (CM: 0.6% yeast extract, 0.6% casein hydrolysate, 1% sucrose, 1.5% agar). Conidiation was examined by harvesting conidia from colonies cultured on rice-bran agar medium (2% rice-polish, 1.5% agar, and pH 6.5) at 26 °C under constant light to promote conidial development.

For pathogenicity assays, conidia were collected from 7-day-old rice-bran medium. Conidial suspensions were adjusted to 1.5–2.0 × 10^5^ conidia/mL in 0.02% Tween solution and sprayed onto three- to four-week-old susceptible rice seedlings (*Oryza sativa* cv. TP309), *Digitaria sanguinalis*, *Setaria viridis*, and *Eleusine indica*. Inoculated plants were incubated in a humid chamber at 25 °C for 24 h and after that moved to another humid chamber with 12 h photoperiod. The plants were examined for disease symptoms at 7 days post inoculation (dpi). For barley (*Hordeum vulgare* cv. Jinchang 1316) and rice sheath inoculation, conidial suspensions (3 × 10^4^ conidia/mL) were injected into barley leaf or rice sheaths and incubated in a dark, humid chamber at 25 °C for 24 and 48 h. The epidermal layers of barley leaf and rice sheath were examined for penetration and proliferation under microscope.

Genomic DNA were extracted using the CTAB extraction method from mycelia cultured in liquid CM medium(CM: 0.6% yeast extract, 0.6% casein hydrolysate, 1% sucrose, 1.5% agar) with 130 rpm shaking at 26 °C for 3 to 4 days. Conidiation was examined by harvesting conidia from 10-day-old mutants and wild type colonies cultured on rice-bran agar medium (2% rice-polish, 1.5% agar, and pH 6.5) at 26 °C under constant light to promote conidial development. Sequencing libraries were prepared using the Illumina Paired-End DNA sample Prep Kit and sequenced by Illumina Hiseq2500 with 50 bp pair-end read length and 500 bp insert size.

### Genome assembly, gene prediction and annotation

The raw data generated from sequencing were evaluated and filtered to eliminate low quality reads with FastQC^56^. Before assembly KmerGenie^57^ was used to predict assembly size. *De novo* sequence assembly were conducted using CLC Genomic Workbench 7.0 with minimum contig length: 500, mismatch cost: 2, insertion cost: 3, deletion cost: 3, length fraction: 0.5, similarity fraction: 0.8. Gene predictions was conducted through a combination of evidence-based prediction by Exonerate^58^ (version 2.2.0) with *M. oryzae* 70-15 genes as reference and *de novo* prediction with Fgenesh from SoftBerry (http://linux1.softberry.com/berry.phtml) with *Magnaporthe* as training organism. All genes predicted from the above approaches were combined by an in-house perl script into a non-redundant set of genes. Functional gene ontologies of genes were predicted by InterproScan version 4.8 (http://www.ebi.ac.uk/interpro/).

Secreted proteins are defined as proteins contains a signal peptide cleavage site, no transmembrane domain after the region signal peptide cleavage site and amino acid length smaller than 400 aa. SignalP 4.1 have been used to predict signal peptide and TMHMM 2.0 used to predict the transmembrane domain^59^,^60^. The present and absent polymorphism (PAV) of secreted proteins was compared using bidirectional blastP (E value < 10^–5^) of amino acid sequences belonging to different isolates.

### SNP calling

All the sequenced reads were aligned to the reference genome *Magnaporthe oryzae* 70-15 with Bowtie2 with default parameters^61^. The SAMtools (version 0.1.19) and Genome Analysis Toolkit, GATK (version 3.3.0) with -genotypeMergeOptions UNIQUIFY, have been used to do SNP calling^62^,^63^,^64^,^65^,^66^. Neighbor-Joining method, bootstrap 100, of MEGA6.0 have been used to construct SNPs and pan genome tree^67^. The genome to genome SNP calling was obtained by the NUCmer of MUMmer^68^, version 3.23, with parameter: -maxmatch -c 100 –p. Principal Component Analysis (PCA) based on 46,530 SNPs obtained from 6 sequenced isolates was conducted by using Tassel 5.0^69^ and plotting with R package Pheatmap. Whole genome collinear analysis was performed by MCscanX^70^.

### Annotation of transposon elements

For the newly sequenced isolates, a transposable elements assembler based on reads K-mer tool, Tedna was used^71^. Also, we constructed a *de novo* repeat library for all isolates using RepeatModeler (version 1.0.8) based on assembled genome with the default parameters, which generated consensus sequences and classification information for each repeat family. Two different results were merged and redundant sequences were removed by blastn and classified with TEclass^72^. RepeatMasker (version 3.3.0) (http://www.repeatmasker.org/) has been used to search for TE in genome with our library^73^. The copy number variation of transposon elements was analyzed based on RepeatMasker results.

### Phylogenomic tree construction and population structure estimated

To construct the phylogenomic tree of sequenced isolates, whole genome protein sequences of *Colletotrichum graminicola*, *Colletotrichum higginsianum*, *Fusarium graminearum*, *Gaeumannomyces graminis*, *Magnaporthe poae* and *Neurospora crassa* were download from Broad Institute of Harvard and MIT (http://www.broadinstitute.org/) and the single copy genes that shared by all the genomes have been selected out by using orthoMCL (version 2.0.9) with E value <10^−5^ and coverage >50%, and then aligned with ClustalW^74^. The phylogenomic tree was constructed using MEGA 6.0 based on the alignments of single-copy ortholog families with Neighbor-Joining method and bootstrap 100.

### Gene family comparison, expansion analysis and duplicated gene detection

Gene family comparison and expansion analysis was based on phylogenomic data obtained by orthoMCL (version 2.0.9) and calculated by CAFE with lambda 0.02, P value < 0.01 and random samples 1000^75^. The gene duplication was detected by blastn, the genes with E < 10^−10^, identity >95% and different location in the genome have been defined as duplicated genes.

### Natural Selection Calculation

The reciprocal best hits (RBH) gene sets obtained by bidirectional blastn (E value < 10^–5^) of different isolates have been paired and aligned with ClustalW^76^,^77^. DNAsp have been used for nucleotide diversity calculation^78^. KaKs Caculator 2.0 have been used to calculate KaKs values with YN model^79^,^80^.

### Database submission

Assembled genomes are available at NCBI under BioProject ID: PRJNA304354.

## Additional Information

**How to cite this article**: Zhong, Z. *et al*. Directional Selection from Host Plants Is a Major Force Driving Host Specificity in *Magnaporthe* Species. *Sci. Rep*. **6**, 25591; doi: 10.1038/srep25591 (2016).

## Supplementary Material

Supplementary Information

Supplementary Table S1

Supplementary Table S2

Supplementary Table S3

Supplementary Table S4

Supplementary Table S5

## Figures and Tables

**Table 1:** Summary of *de novo* genome assembly of *Magnaporthe* isolates from different host plants.

Isolate	DS9461	DS0505	EI9604	EI9411	SV9623	SV9610
Assembled contigs	2,201	1,984	1,347	1,661	1,514	1,548
Genome size (Mb)	42.5	42.7	39.7	38.5	37.6	37.5
GC content (%)	48.7	48.6	50.2	50.6	51	51
N50 length (bp)	63,753	73,656	100,075	81,416	145,603	154,236
Max contig length (bp)	349,171	501,225	472,660	501,659	585,182	948,369
Number of genes	12,914	12,975	14,237	14,008	13,933	13,847
Average gene length	1,622	1,626	1,452	1,452	1,439	1,467
Coding region of assembly (%)	49.3	49.4	52.1	52.9	53.3	54.2
Host plant	*Digitaria sanguinalis*	*Digitaria sanguinalis*	*Eleusine indica*	*Eleusine indica*	*Setaria viridis*	*Setaria viridis*

**Table 2:** Nucleotide diversity and present and absent polymorphisms of avirulent genes in sequenced isolates.

AVR gene	Nucleotide Diversity	DS9461	DS0505	EI9411	EI9604	SV9610	SV9623	98–06	KJ201	P131	Y34
*AvrPita*	0.01126	P	P	A	A	P	P	P	P	P	P
*AvrPib*	0.01382	A	P	P	A	P	P	P	P	P	P
*PWL2*	0.00963	P	P	A	A	A	A	P	P	A	P
*PWL1*	0.01126	A	A	P	A	A	A	A	A	A	A
*AvrCO39*	0.00444	A	A	P	P	P	P	A	A	A	A
*AvrPia*	0	A	A	A	A	A	A	A	A	P	P
*AvrPii*	0	A	A	A	P	A	A	A	A	P	A
*AvrPik*	0.00439	A	A	A	A	A	A	P	P	A	P
*AvrPiz-t*	0.00832	A	A	P	P	P	P	P	P	P	P
*AvrPi9*	0.00455	A	A	P	P	P	P	P	P	P	P

P indicate genes that are present in the subject genome, A indicate genes that are absent in the subject genome.
