# Supplementary material for: Directional Selection from Host Plants Is a Major Force Driving Host Specificity in Magnaporthe Species
Source: Sci Rep. 2016 May 6;6:25591. doi: 10.1038/srep25591 (PMC4858695; doi:10.1038/srep25591)
Supplement: Supplementary Information [file srep25591-s1.doc]

**Directional Selection from Host Plants Is a Major Force Driving Host Specificity in *Magnaporthe* Species**

Zhenhui Zhong^a,b#^, Justice Norvienyeku^a,b#^, Meilian Chen^a,b#^, Jiandong Bao^a,b#^, Lianyu Lin^a,b^, Liqiong Chen^b,c^, Yahong Lin^b,c^, Xiaoxian Wu^a,b^, Zena Cai^a,b^, Qi Zhang^a,b^, Xiaoye Lin^a,b^, Yonghe Hong^b,c^, Jun Huang^a,b^, Linghong Xu^a,b^, Honghong Zhang^a,b^, Long Chen^a,b^, Wei Tang^b,c^, Huakun Zheng^d^, Xiaofeng Chen^a,b^, Yanli Wang^f^, Bi Lian^a,b^, Liangsheng Zhang^g^, Haibao Tang^g^, Guodong Lu^a,b^, Daniel J. Ebbole^a,h^ , Baohua Wang^a,b,c*^, Zonghua Wang^a,b,c*^

^a^Fujian-Taiwan Joint Center for Ecological Control of Crop Pests, Fujian Agriculture and Forestry University, Fuzhou, 350002, China.

^b^Fujian University Key Laboratory for Functional Genomics of Plant Fungal Pathogens, Fujian Agriculture and Forestry University, Fuzhou, 350002, China.

^c^Fujian Province Key Laboratory of Pathogenic Fungi and Mycotoxins, Fujian Agriculture and Forestry University, Fuzhou, 350002, China.

^d^Haixia Institute of Science and Technology (HIST), Basic Forestry and Proteomics Research Center, Fujian Agriculture and Forestry University, Fuzhou, 350002, China.

^f^State Key Laboratory Breeding Base for Zhejiang Sustainable Pest and Disease Control, Institute of Plant Protection Microbiology, Zhejiang Academy of Agricultural Sciences, Hangzhou, 310021, China.

^g^Haixia Institute of Science and Technology (HIST), Center for Genomics and Biotechnology, Fujian Agriculture and Forestry University, Fuzhou, 350002, China.

^h^Department of Plant Pathology and Microbiology, Texas A&M University, College Station, TX, USA.

# These authors contributed to this work equally.

*** To whom correspondence should be addressed. Email: [wangzh@fafu.edu.cn](mailto:wangzh@fafu.edu.cn) or [wbaohua@fafu.edu.cn](mailto:wbaohua@fafu.edu.cn).

**Supporting Information**

**
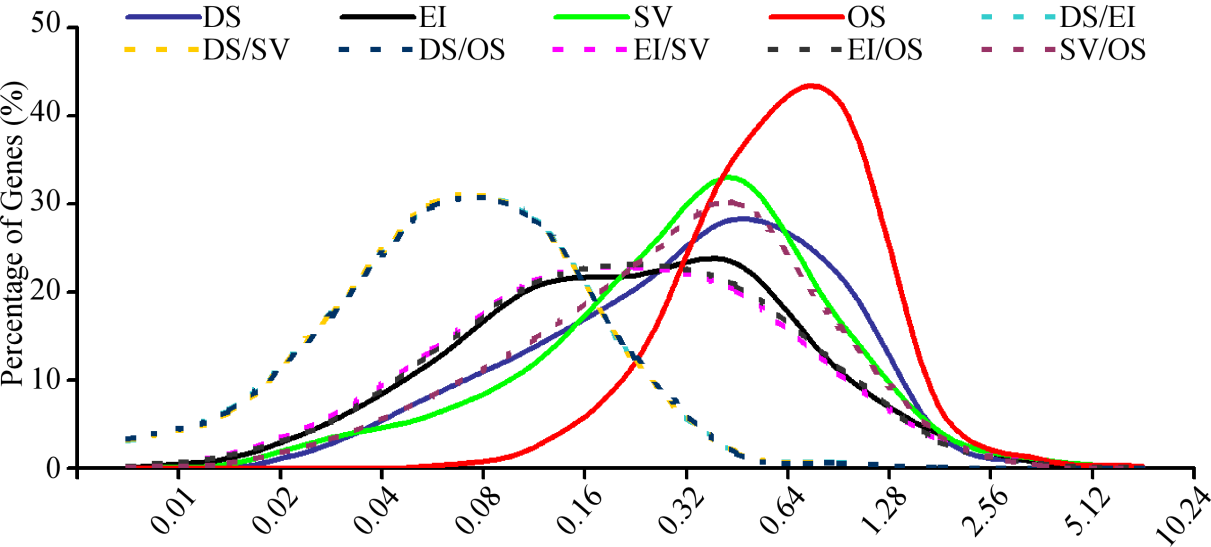
**

**Supplementary Figure S1.** Comparison of the percentage of genes experienced different level of natural selection (KaKs) inter- and intra-groups. X-axis represents values of KaKs and Y-axis represents percentage of genes with corresponding KaKs value. DS, *D. sanguinalis* isolates, EI, *Eleusine indica* isolates, SV, *Setaria viridis* isolates and OS, *Oryza sativa* isolates.


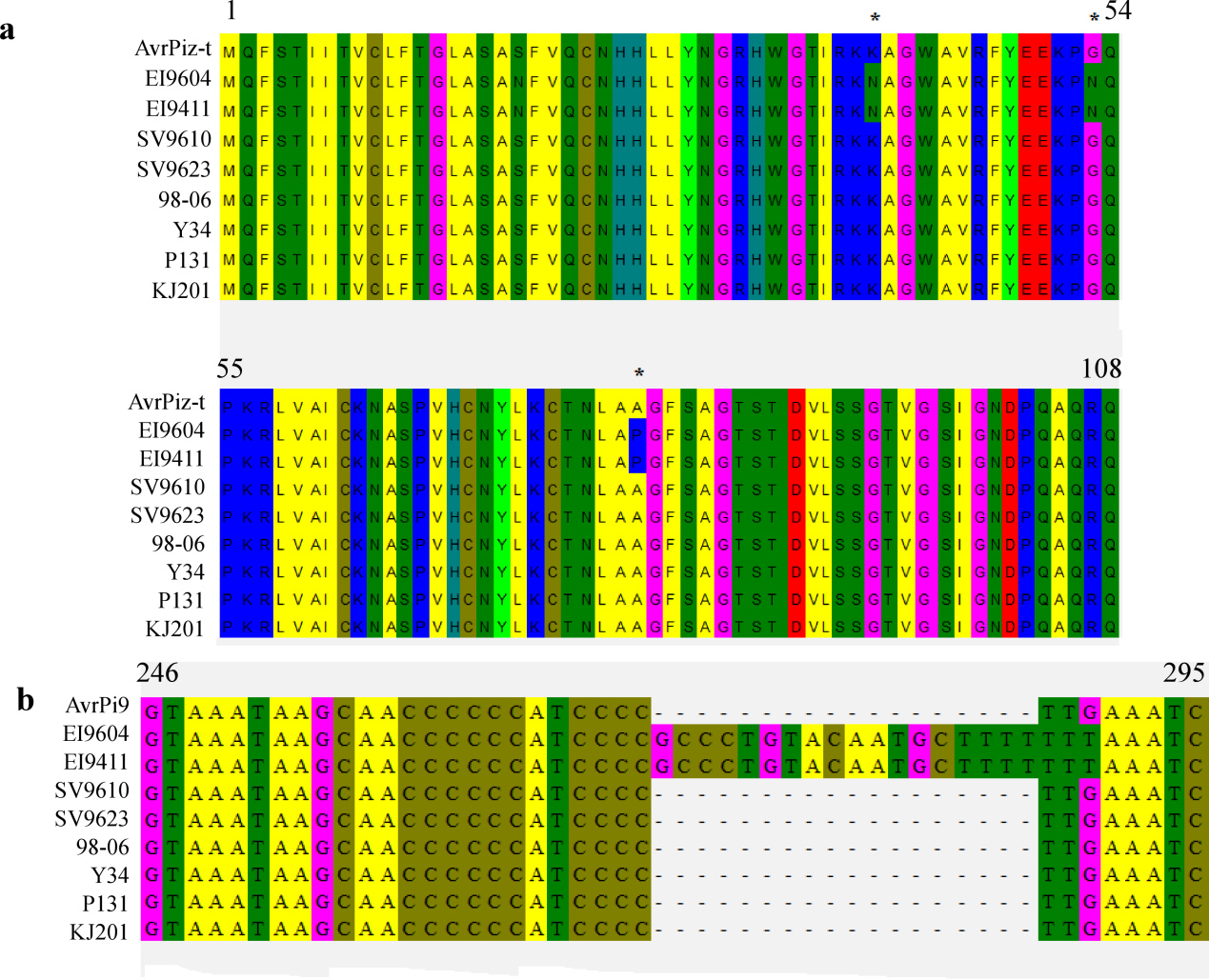


**Supplementary Figure S2.** Alignment of AvrPiz-t and AvrPi9. **(a)** Alignment of amino acid sequence of *AvrPiz-t* in different isolates. **(b)** Alignment of nucleotide sequence of *AvrPi9* in different isolates.
